# Results from a large targeted screening program for alpha-1-antitrypsin deficiency: 2003 - 2015

**DOI:** 10.1186/s13023-016-0453-8

**Published:** 2016-06-10

**Authors:** Timm Greulich, Christoph Nell, Christian Herr, Claus Vogelmeier, Viktor Kotke, Stefan Wiedmann, Marion Wencker, Robert Bals, Andreas Rembert Koczulla

**Affiliations:** Department of Medicine, Pulmonary and Critical Care Medicine, University Medical Center Giessen and Marburg, Marburg, Germany; Philipps-University Marburg, Member of the German Center for Lung Research (DZL), Baldingerstraße, 35043 Marburg, Germany; Department of Internal Medicine V, Pulmonology, Allergology, Respiratory and Environmental Medicine, Saarland University Hospital, 66421 Homburg/Saar, Germany; Department of Pneumology, University Hospital Essen, Ruhrlandklinik, 45239 Essen, Germany

**Keywords:** Alpha 1-antitrypsin deficiency, Asthma, Bronchiectasis, Chronic obstructive pulmonary disease, Targeted screening

## Abstract

**Background:**

Alpha-1-antitrypsin deficiency (AATD) is an autosomal codominant inherited disease that is significantly underdiagnosed. We have previously shown that the combination of an awareness campaign with the offer of free diagnostic testing results in the detection of a relevant number of severely deficient AATD patients. The present study provides an update on the results of our targeted screening program (German AAT laboratory, University of Marburg) covering a period from August 2003 to May 2015.

**Methods:**

Diagnostic AATD detection test kits were offered free of charge. Dried blood samples were sent to our laboratory and used for the semiquantitative measurement of the AAT-level (nephelometry) and the detection of the S- or Z-allele (PCR). Isoelectric focusing was performed when either of the initial tests was indicative for at least one mutation. Besides, we evaluated the impact of additional screening efforts and the changes of the detection rate over time, and analysed the relevance of clinical parameters in the prediction of severe AATD.

**Results:**

Between 2003 and 2015, 18,638 testing kits were analysed. 6919 (37.12 %) carried at least one mutation. Of those, we identified 1835 patients with severe AATD (9.82 % of the total test population) including 194 individuals with rare genotypes. Test initiatives offered to an unselected population resulted in a dramatically decreased detection rate. Among clinical characteristics, a history of COPD, emphysema, and bronchiectasis were significant predictors for Pi*ZZ, whereas a history of asthma, cough and phlegm were predictors of not carrying the genotype Pi*ZZ.

**Conclusion:**

A targeted screening program, combining measures to increase awareness with cost-free diagnostic testing, resulted in a high rate of AATD detection. The clinical data suggest that testing should be primarily offered to patients with COPD, emphysema, and/or bronchiectasis.

**Electronic supplementary material:**

The online version of this article (doi:10.1186/s13023-016-0453-8) contains supplementary material, which is available to authorized users.

## Background and objectives

Alpha 1-antitrypsin (AAT) deficiency (AATD), one of the most prevalent inherited disorders in populations of European descent, is characterized by abnormally low serum levels of AAT [1]. The leading disease manifestations in AATD patients are chronic obstructive pulmonary disease (COPD) with early onset emphysema, particularly in individuals who smoke. Further associated diseases comprise liver disease, cutaneous panniculitis, bronchiectasis, vasculitis, Wegener’s granulomatosis and lung cancer [2–6].

To date, more than 100 genetic variants of the AAT gene (*SERPINA1*) have been identified [7]. The proteinase inhibitor (PI) MM genotype is the predominant phenotype amongst those considered as normal whereas most individuals with severe AATD are homozygous PI*ZZ (or Pi*Z0, Pi*00) showing a significantly reduced AAT serum concentration [8, 9]. Reports based on genetic epidemiologic surveys estimate that about 180.000 individuals worldwide carry the phenotype Pi*ZZ [10]. In contrast, recent data from the European Alpha-1 international registry (AIR) indicate 4.758 registered individuals with severe deficiency implying that only 3,8 % of the severely affected individuals have been identified [11]. Besides, several studies have demonstrated substantial delays in the diagnosis of AATD [12, 13]. On average, AATD patients experience a diagnostic delay of 6 years and have to consult three physicians until the diagnosis is established [14]. The average age of diagnosis for AATD patients is 45.5 years, but one third is not diagnosed until their fifties [12].

ATS (American Thoracic Society)/ERS (European Respiratory Society) and WHO provided recommendations for genetic testing in order to enhance the detection of severe AATD patients [1, 15]. Briefly, according to the WHO all individuals with COPD, emphysema or asthma or with a family history of the disorder should be screened once for AATD using a quantitative test [15]. ATS and ERS guidelines recommend testing for all patients with COPD, emphysema, or asthma with irreversible airflow obstruction [1]. Interventions undertaken to identify AATD individuals include screening and targeted detection studies [16]. Screening studies investigate unselected groups without suspicion of having AATD whereas targeted (case-finding) studies focus on individuals with a high risk for AATD. As a result, targeted approaches achieve much higher rates of detection of AATD: Several studies reported detection rate of Pi*ZZ ranging from 0.37 to 12 % [17], depending on the location, the inclusion criteria and the number of individuals investigated.

In the present paper, we provide an update on the results of our targeted screening program (German AAT laboratory, University of Marburg) covering the period of August 2003 to September 2015 and including the results of 18,683 test kits.

## Methods

The present study gives an update of the German targeted screening program which has been already described in detail [18]. Briefly, it combines a diagnostic service (offering a cost-free diagnostic test concerning AATD) with the aim to increase the awareness of physicians with regard to potential AATD individuals. The diagnostic approach combines the initial measurement of the AAT serum level and a targeted PCR to detect the two most common mutations (Pi*S and Pi*Z) with further testing (genotyping, gene sequencing) if necessary [18].

Furthermore, “screening events” have been carried out: In these events (most often performed after a meeting of patient organizations), all individuals were invited to undergo testing, regardless of presence/absence of respiratory symptoms or a serum level. All patients gave informed consent for genetic testing. Because the study reflects a retrospective analysis of routine data, an ethics approval was not necessary.

### Diagnostic approach

All samples underwent nephelometry for determination of the AAT-level and PCR for the detection of the S- and Z-allele, which was eventually followed by isoelectic focusing and gene sequencing. The results of PCR and IEF were read out and combined by two independent readers. Details regarding the laboratory methods used can be found in the online supplement (Additional file 1: Details on laboratory methods) and have been described before [18].

The results were reported to the medical doctor who had sent the testing kit. All procedures were quality controlled and based on standard operating procedures (SOPs). All laboratory procedures included appropriate negative and positive controls. The AATD Laboratory Marburg participates successfully in interlaboratory comparisons.

### Data storage and analysis

The test results were entered into a Microsoft Access database. To compare detection rates between the overall results and screening events only, Fisher’s exact test was used. To analyse the effect of the presence/absence of symptoms on the pre-test probability to identify Pi*ZZ, a multiple logistic regression analysis was performed with the following parameters: Pi*ZZ as being the dependent variable and clinical characteristics that were recorded on the kit (acute bronchitis, asthma, bronchiectasis, COPD, dyspnoea on exertion, dyspnoea attacks, emphysema, chronic bronchitis, cough, sputum, wheezing) being the independent variables. All analyses were carried out using SPSS Version 20 (IBM, Ehningen, Germany), and GraphPad Prism Version 6 (GraphPad Software, Inc., La Jolla, USA).

## Results

**Fig. 1:**
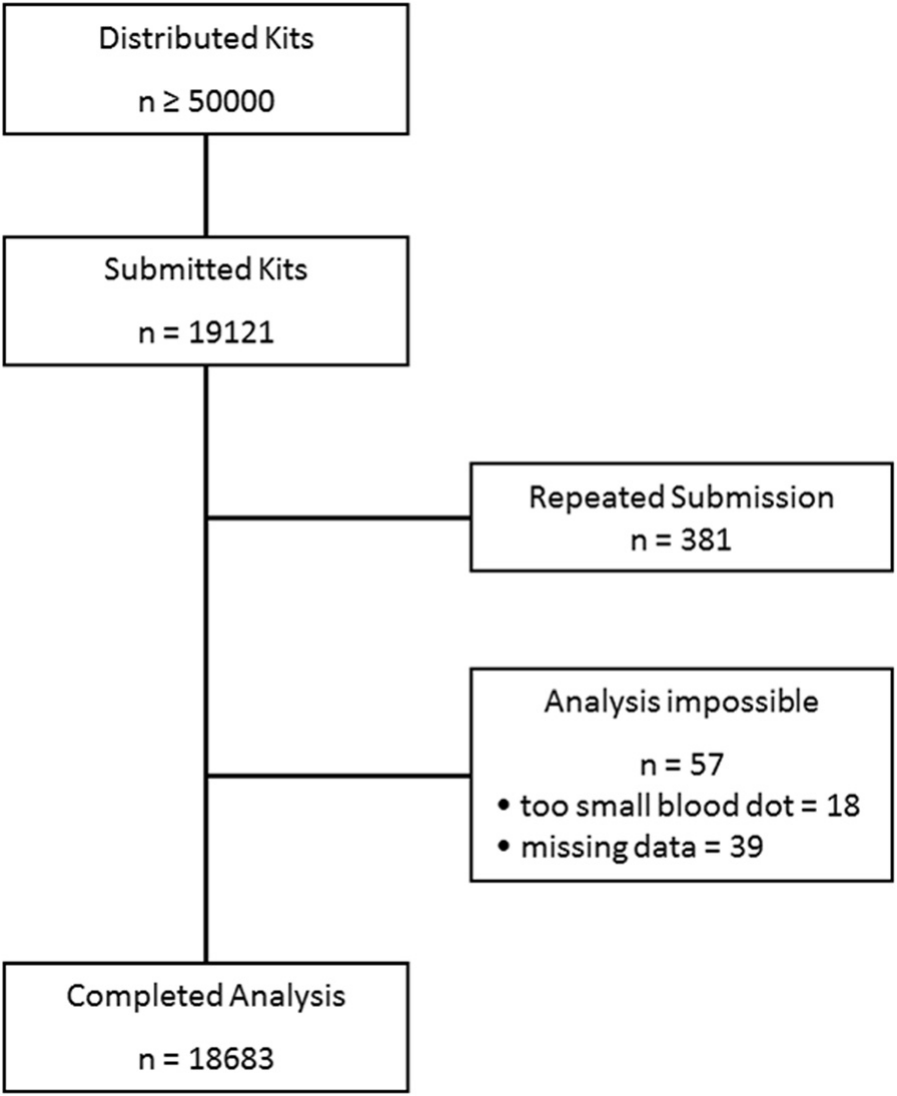
Over 50,000 test kits have been distributed. Kits from patients that had already been tested (*n* = 381) and kits that were not analyzable (*n* = 57) were removed from the final analysis

From August 2003 to September 2015, >50,000 test kits were sent out on request. A total number of 19,121 kits were received in our laboratory for analysis (500 – 2500 per year); 57 were not analyzable (not enough material: *n* = 18; missing data: *n* = 39) and 381 had been submitted more than once. Therefore, our final analysis includes 18,683 samples (Fig. 1).

**Fig. 2:**
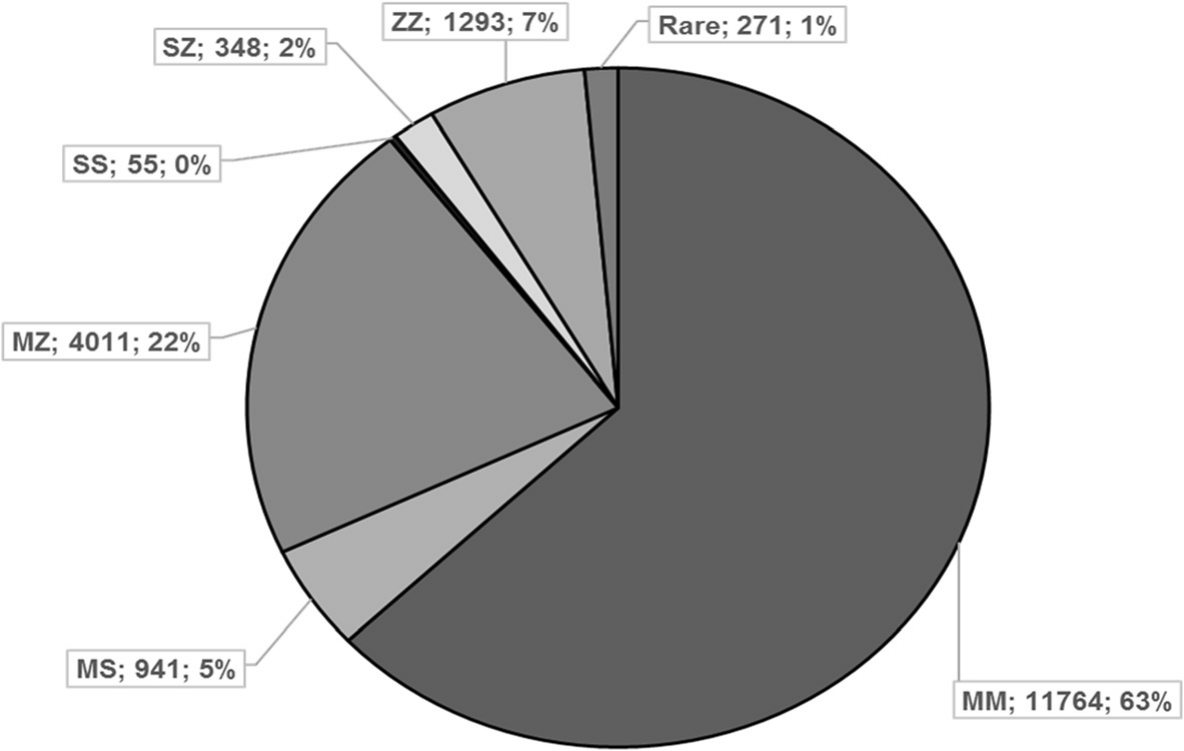
Results from 18,683 kits that have been analysed successfully within the first 12 years of the laboratory (August 2003 to September 2015)

We identified 6919 patients with at least one deficiency allele (37.12 %) including 271 individuals with rare genotypes. In descending order of frequency, we have diagnosed the following genotypes (Fig. 2): Pi*MM (11764; 62.97 %), Pi*MZ (4011; 21.47 %), Pi*ZZ (1293; 6.92 %), Pi*MS (941; 5.04 %), Pi*SZ (348; 1.86 %), Pi*SS (55; 0.29 %). In samples that underwent gene sequencing (results not clear after standard PCR and IEF), we found 271 rare genotypes.

Compared to the overall results of the laboratory, the screening events yielded significantly less positive results (at least one deficiency allele) (Table 1).

**Table 1:** Results of screening events (*n* = 1048) compared to the results of the entire test population (*n* = 17,635). Displayed are percentage numbers of the laboratory population (left column) and the screening event population, respectively (right column)

	Laboratory	Screening	*p*-value
	Population	Event	(Fisher-Test)
MM	63.48	88.02	<0.001
MS	5.05	4.56	0.62
MZ	20.38	5.13	<0.001
SZ	1.88	0.16	<0.001
SS	0.31	0.08	0.25
ZZ	7.09	0.07	<0.001
Rare	1.44	0	<0.01

After an initial drop of the detection rate (% samples Pi*ZZ in a given time period), the rate increased from 2008/09 over the following years. Exploring potential reasons for that we described the three most frequent clinical characteristics that led to screening (Table 2). We found that time periods associated with high detection rates included either emphysema or COPD while the time periods with the lowest detection rates (2008/09 and 2010/11) did not (Table 2).

**Table 2:** Displayed are the detection rates (Pi*ZZ) of different time periods, the characteristics of the population screened at that time (age in years; mean ± standard deviation) and the three most frequent clinical characteristics that led to the test initiation. Numbers do not add up to 18,683, because for some individuals the gender was not recorded and could not be deducted by the name

	Detection rate [%]	Age [years]	Male/female	Most frequent clinical characteristics
2003/04/05	8.65	39.8 ± 21.9	1105/887	1. Cough
				2. Emphysema
				3. Wheezing
2006/07	4.04	40.4 ± 21.7	1527/1399	1. Dyspnoea on exertion
				2. Cough
				3. Wheezing
2008/09	4.25	48.9 ± 21.6	1973/1791	1. Dyspnoea on exertion
				2. Cough
				3. Phlegm
2010/11	4.98	48.2 ± 20.3	2127/1888	1. Dyspnoea on exertion
				2. Cough
				3. Phlegm
2012/13	7.91	47.9 ± 19.1	1808/1661	1. Dyspnoea on exertion
				2. Cough
				3. COPD
2014/15	11.26	48.5 ± 18.8	1280/1139	1. Dyspnoea on exertion
				2. COPD
				3. Cough
All	6.85	45.9 ± 20.8	9820/8765	1. Dyspnoea on exertion
				2. Cough
				3. COPD

**Fig. 3:**
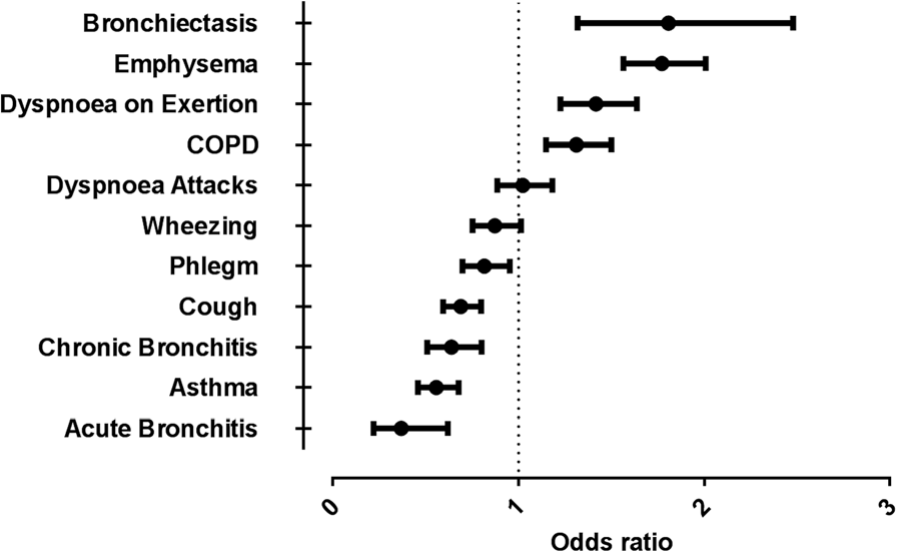
Clinical characteristics predictive for the genotype Pi*ZZ (multivariate logistic regression analysis)

In a multivariate logistic regression analysis of clinical features of the individuals that underwent testing, we found emphysema, bronchiectasis and COPD being the strongest predictors of Pi*ZZ (Fig. 3). Acute bronchitis, chronic bronchitis, cough, wheezing, and phlegm were associated with decreased likelihood of detecting Pi*ZZ (Fig. 3).

## Discussion

From 2003 to 2015, we analyzed 18,683 DBS samples with PCR and nephelometry in our AAT diagnostic laboratory in Marburg, Germany. In 1835 (9.82 %) of these we identified an individual with severe AATD. Our results correspond to those of other laboratories that have initiated targeted detection regarding AATD and focus on test populations showing clinical features of the disease.

The Italian program distributing DBS AATD tests to hospitals and clinics suggested testing of individuals with early-onset emphysema and familial cluster of COPD, with a plasma AAT level <80 mg/dl or first degree relatives of subjects with known AATD (including Pi*MZ heterozygosity) [19, 20]. Of 2127 samples, 10.6 % presented with severe deficiency (Pi*ZZ, Pi*SZ and rare genotypes). Carroll et al. screened 3000 individuals as part of the Irish National Targeted Detection Programme and included subjects with COPD, non-responsive asthma, cryptogenic liver disease, first degree relatives of AATD patients and individuals with reduced serum AAT levels [21]. 3.1 % were found to have a severe deficiency. Corda et al. chose an extremely selected study population displaying specific characteristics of AATD in order to perform a targeted screening study and identified 15.8 % severely deficient phenotypes (12 % Pi*ZZ) in a rather small study sample (*n* = 285) [22]. On the other hand, screening studies including non-selected patients with COPD or other respiratory clinical syndromes were not able to detect severe AATD phenotypes in more than 0.5 % of the study population [23–25]. These data once more underline the importance of careful selection regarding individuals suspected to have AATD when performing a targeted screening program. To our knowledge, our analysis represents the as yet biggest targeted screening cohort in Europe (*n* = 18,683). According to estimates of the frequency of Pi*ZZ in Germany [26], 8003 individuals would be expected. Having detected 1293 individuals (16.15 %) is a higher percentage than has been reported in many other countries of the world as pointed out by a recent review [27].

Comparing our data to those previously published [18] we were able to document stability of the results over time which might derive from the continuous preselection of the study population, the high number of analyzed samples and the experience of the Marburg laboratory with applying diagnostic methods concerning AATD. While the results remained rather stable comparing the first published analysis with the recent one, it has to be acknowledged that there was a drop in the detection rate during 2008 – 2011 (Table 2). The increase after that may – at least in part – be explained by COPD becoming a more prominent cause to send samples to our laboratory, reflecting a population being more likely to suffer from severe AATD (Table 2). Regarding the relatively high detection rate in 2003 – 2005, younger patients with emphysema have been tested, again a population more likely to carry a severe deficiency *SERPINA1* genotype (Table 2).

By using our test algorithm, we intended to investigate especially those patients who showed low concentrations of AAT levels in order to prevent unnecessary diagnostic steps and save costs. On the other hand, heterozygous carriers of *SERPINA1* mutations may have been missed because the determination of the AAT level precedes sending of tests to our laboratory. Ferrarotti et al. (University of Pavia) perform genotyping (by PCR-RFLP using TaqI as the restriction enzyme) at the first stage of their algorithm simultaneously with the quantitative measurement of AAT-levels [28], resulting in a higher rate of testing in samples with normal AAT-levels. Furthermore, the testing algorithm used by the laboratory of the university in Pavia allows consecutive sequence analyses if the results of the phenotyping do not produce clear evidence about deficiency phenotypes whereas our laboratory repeats nephelometry, PCR and IEF in whole blood samples of individuals with conflicting results from DBS analyses before performing additional DNA sequencing.

We detected a relatively high number of rare genotypes other than Pi*ZZ or Pi*SZ among the individuals with severe AATD (194/1835 = 10.57 %) which is more than, for example, the rate of 1.7 % reported by the NHLBI Registry [29], and comparable with results published by Ferrarotti et al. who recorded an 11 % prevalence of subjects with severe AATD carrying rare variants [30]. In the light of a new test that identifies individuals with a Z-allele but would not detect individuals with severe deficiency without one or two Z alleles [31] it is reassuring to notice that only very few individuals (22 S/rare, 17 rare/rare) would be missed by this test.

The majority of kits was returned by pulmonologists reflecting the pulmonary symptoms most patients with symptomatic AATD suffer. The low number of gastroenterologists who returned DBS to our laboratory might be explained by the fact that liver problems deriving from an underlying AATD occur directly after birth. During this phase of life testing will be usually requested by the pediatricians. Evaluating targeted AATD screening programs should also include the aspect of cost-effectiveness. In fact, it has been shown that case-finding in populations in which AATD is suspected in reasonable frequency (e.g. patients with known COPD) could satisfy cost-effectiveness criteria [32]. In line with this, our testing algorithm reveals its potential to save costs by applying phenotyping (<50 % IEF) and sequencing (~1 %) in a limited number of individuals.

With regard to clinical characteristics that would be predictive for Pi*ZZ we confirmed earlier reports of COPD and emphysema being important predictors. The fact that bronchiectasis was a strong predictor for Pi*ZZ is interesting, because earlier reports gave conflicting results [33–35]. Our results would support screening for AATD in this patient group that is increasingly recognized [36]. Asthma and acute bronchitis were the strongest clinical predictors of not carrying a homozygous deficiency. This confirms studies that did not report an increased prevalence of homozygous AATD in asthmatic patients [37, 38].

Concerning potential limitations of our screening program we acknowledge that targeting selected (symptomatic) individuals might result in missing asymptomatic subjects with severe AATD. This has been suggested by several studies which investigated the percentage of Pi*ZZ individuals with clinical symptoms. A significant proportion of homozygote Pi*ZZ subjects do neither develop emphysema [39] nor COPD [40]. Besides, we have only limited information about the patients, their clinical characteristics or serum AAT levels. Reporting symptoms (beyond the clinical characteristics that have been asked for), lung function, smoking habits and family screening would have been helpful in characterizing AATD individuals.

## Conclusions

In conclusion, we have shown that our targeted screening program, combining an awareness campaign with cost-free diagnostic testing, resulted in a high rate of AATD detection, especially in patients with COPD, emphysema, and bronchiectasis. In the context that AATD is still an under-diagnosed disease and that recognition may entail important implications (e.g. smoking avoidance, family testing, augmentation therapy), targeted screening approaches should be continued.

## Ethics approval and consent to participate

All patients gave informed consent for genetic testing. Because the study reflects a retrospective analysis of routine data, an ethics approval was not necessary.

## Consent for publication

Not applicable.

## Availability of data and materials

Data will not be shared. The data are subjects to further scientific analyses that may be published in the future.

## Additional file

Additional file 1: Details on laboratory methods. (DOCX 12 kb)

## Abbreviations

AATD: Alpha-1-antitrypsin deficiency
ATS: American Thoracic Society
COPD: chronic obstructive pulmonary disease
DBS: dried blood spot
DNA: deoxyribonucleic acid
ERS: European Respiratory Society
IEF: isoelectric focusing
NHLBI: National Heart, Lung and Blood Institute
PCR: polymerase chain reaction
RFLP: restriction (Enzyme) fragment length polymorphism
SOP: standard operating procedure
