# Supplementary material for: Results from a large targeted screening program for alpha-1-antitrypsin deficiency: 2003 - 2015
Source: Orphanet J Rare Dis. 2016 Jun 10;11:75. doi: 10.1186/s13023-016-0453-8 (PMC4901499; doi:10.1186/s13023-016-0453-8)
Supplement: Additional file 1: — Details on laboratory methods. (DOCX 12 kb) [file 13023_2016_453_MOESM1_ESM.docx]

**Additional file 1: Details on laboratory methods**

Nephelometry: The AAT amount of a circular disk of the DBS (4,5 mm) was deter-mined (Nephelometer Analyzer 2, Dade Behring, Frankfurt, Germany) as described before [18].

PCR: We amplified two genomic regions of the Z- and S-allele and performed a sub-sequent digestion with a restriction enzyme that results in a genotype specific diges-tion pattern. The presence of the Z- or S-allele was determined by comparing the typi-cal bands to standard controls. Details have been described before [18].

Isoelectric Focussing: Isoelectric focussing (IEF) was performed if mutations were suspected (low AAT serum levels as reported by the sending physician, low nephelo-metric values or presence of Z- or S-band in the PCR). Details have been described before [18]. The results were read out by two blinded observers.

Sequencing: If the results of the PCR and IEF yielded inconsistent results or if there were indications for a rare or unknown genotype, we requested serum and EDTA-blood for gene sequencing. Data on newly identified mutations on the SERPINA1 gene will be published separately.
